# Supplementary material for: The impact of Worms and Ladders, an innovative health educational board game on Soil-Transmitted Helminthiasis control in Abeokuta, Southwest Nigeria
Source: PLoS Negl Trop Dis. 2020 Sep 25;14(9):e0008486. doi: 10.1371/journal.pntd.0008486 (PMC7549763; doi:10.1371/journal.pntd.0008486)
Supplement: Attachment — Submitted filename: Response to reviewers comment_3.docx [file pntd.0008486.s002.docx]

**Reviewer 3**

Reviewer's Responses to Questions

**Key Review Criteria Required for Acceptance?**

As you describe the new analyses required for acceptance, please consider the following:

**Methods**

-Are the objectives of the study clearly articulated with a clear testable hypothesis stated?

-Is the study design appropriate to address the stated objectives?

-Is the population clearly described and appropriate for the hypothesis being tested?

-Is the sample size sufficient to ensure adequate power to address the hypothesis being tested?

-Were correct statistical analysis used to support conclusions?

-Are there concerns about ethical or regulatory requirements being met?.

Reviewer #3:

C1: The description of the intervention is quite basic. A more complete description of the messages used, as well as the auxiliary discussion with teachers is warranted.

R1: Thank you for this valuable comment. We have improved out text to provide a more complete description on how the messages were formed, and what kind of messages was included. However, there was no auxiliary discussion with teachers during development of the game.

Line 188-211:

**Designing the “Worms and LaddersTM” game**

As an initial step during the development of an intervention tool, a consultation meeting among investigators was held to identify risk factors for STH infections. The risk factors for STH were considered through desk-review of previous epidemiological studies conducted among school-aged children. Since studies have shown that poor sanitary conditions and personal hygiene such as not wearing shoes or slippers, indiscriminate defecation, not washing hands before eating and/or after using the toilet, lack of toilet facilities and dirty environment predisposes children to STH infections (Bethony *et al*., 2006; Ekpo *et al*., 2008; Ekpo *et al*., 2012; Halpenny *et al*., 2013). The health educational messages were then formed in response to each of the identified risk factor. The messages were presented in simple forms to ensure proper understanding. Ten major health educational messages developed were; washing of hands before eating, washing hands with soap after using the toilet, not playing with soil, wearing of slippers or shoes when going outside, avoiding open (indiscriminate) defecation, washing of fruits and vegetables before consumption, covering of food from flies, keeping fingernails clean and cut, not picking food from the ground, and cleaning the environment.

Furthermore, since the target population for the tool is school-aged children, the team adopted the use of visual aids, as it is one of the strongest tool used in communicating messages; particularly when accompanied with interactive methods (Yazachew and Alem, 2004). In addition, the team considered deploying this tool in a way that facilitates group discussion and demonstration. In consideration of all these, a board game was selected as a means to deliver the health education messages to the school children. Therefore, a board game named “Worms and Ladders^TM^” was developed to teach the school-aged children about transmission, symptoms, treatment, prevention and control of STH infections. The game was piloted in a school setting to identify areas that needs to be worked upon to enhance clarity and facilitate understanding. The pilot study was implemented in an area different from where the study schools were selected.

C2: For the intervention, I would like to see some discussion about the merits of knowledge-based education as a driver of behavior change. I am surprised that knowledge alone would be sufficient to get children to change their behavior without, for example, improvements in access to water and soap or demonstrations of proper HW practice. Perhaps the authors need to provide a bit more context for the study site, since this approach may not work in areas that are more resource/water scarce.

R2: We have modified our introductory text to include more information about the merits of knowledge based education as a driver of behavior change.

Line 89- 103: Health education strategy is economical as it reduces the cost of deworming, increases the level of overall health knowledge and acceptability of deworming interventions (Luong, 2003; Albonico *et al*., 2006) and it also enhances increased understanding of effective health practices (Hotez *et al*., 2006). The inclusion of a health education strategy into school-based deworming programmes has been recommended based on the rationale that exposure to STH infections would be reduced, and re-infection delayed through improved knowledge and behavioural changes (WHO, 2003; Mascarini-Serra, 2011). There is plethora of evidence reinforcing the aforementioned theory on the significant role health educational activities have on positive behavioural change [15-18]. For instance, Bieri and colleagues [13] in China used a video-based health education package to prevent new STH infections after treatment. Similarly, Ejike and colleagues [14] in Nigeria also reported significant improvement in the knowledge, attitudes and practices as regards risk behaviour, prevention and control of schistosomiasis among children who played a health educational board game. Health education thus plays a major role in helminths disease prevention, by decreasing exposure risk through behavioural change, and in increasing health-seeking behaviour.

We have also provided additional information on the context for the study site under our study design and selection of schools

Line 121-138:

***Study design and selection of schools***

This study employed a Randomized Control Trial (RCT) design involving six (6) public primary schools selected out of the 49 public primary schools in the study area. As an initial step, the forty-nine schools in the study area were first stratified into two clusters based on existing geo-political zones, proximities to common boundaries and road networks (Figure 1). Cluster 1 (C1) has a total of 21 schools and cluster 2 has a total of 28 schools. To have an ample sampling frame, six schools were randomly selected, at first using balloting from each cluster. Afterwards, three schools were selected randomly out of the six schools in each cluster using the second round of balloting. Preliminary visits were made to selected schools to document cofounding factors such as the presence of UNICEF-assisted WASH intervention programmes. Schools with UNICEF-assisted programme were replaced purposively with one of the three remaining schools in the sampling frame. The selected schools were blindly assigned to two treatments; Treatment 1 (“Worms and Ladders^TM^” game); and Treatment 2 (“Snakes and Ladders” game) by another researcher who did not partake in the selection procedures. Schools within the intervention group are those who received the newly designed “Worms and Ladders^TM^” game (Figure 2) while those in the control group received the common “Snakes and Ladders” game (Figure 3). The study was conducted between March and October 2015, and it involved 5 distinct phases; baseline, deworming, intervention, three and six-month post intervention follow-up phases.

C3: There is no sample size calculation included.

R3: We have modified our text to include sample size determination method.

Line 158-170:

***Sample size determination and selection of study participants***

The sample size for this study was determined using the formula; $\boldsymbol{n}_{\boldsymbol{s}}\boldsymbol{=}\frac{\boldsymbol{N}}{\boldsymbol{1+}\boldsymbol{Ne}^{\boldsymbol{2}}}$, as described by Yamane (1967) where n_s_= sample size, N= total study population and e = marginal error. As an initial step, enrollment registers for selected schools was reviewed to obtain the total number of school-aged children. The enrollment figure from selected schools was summed up to obtain a total population (N) of 3801 school-aged children. In determining the sample size, a marginal error (e) of 5% was considered at 95% level of confidence. The minimum sample size determined therefore was 362 i.e. an average of 60 school-aged children per selected school. Selection of pupils in each school was carried out after stratification by class grade from Primary 1 to 6. Children from each class grade were voluntarily invited to participate and only those who consented were recruited into the study. Finally, a total of 372 school-aged children were recruited from the six schools. However, in most schools the number of consenting children varied by class grade hence giving roughly an unequal number of pupils recruited.

C4: There is no description of how students were selected within the schools and the types of surveys that were conducted and how (there is a quote from a student in line 345).

R4: We have included how students were selected with schools in between Line 158-170 (see response R3)

We have also provided more detailed information on the type of survey conducted (i.e KAP and FGD) KAP (lines 240-246)

Children’s knowledge, attitude and practices (KAP) towards STH infections were also assessed using structured questionnaires and focus group discussions on three different occasions during the life-time of the study i.e. baseline, follow-up (3 months) and end-line (6-months). The children were interviewed individually in the head-teachers’ office, within the school premises. Where necessary, the interviews were conducted in the native Yoruba language.

Group discussions (Line 270-277)

**Group Discussion (GD)**

During the monitoring and follow-up period, players were invited to participate in a group discussion on the “Worms and Ladders^TM^ ” game. Discussions were among mixed group of boys and girls, and was conducted during and after playing the games. All discussions including impact of the game of players attitude and behaviour, players understanding and interpretation of the health education messages and symbols was recorded, coded and extracted under different themes during analysis. The discussants were informed about the recordings and encouraged to be as free as possible.

C5:Was the analysis conducted to account for clustering at a school-level?

R5: Analysis took care of clustering at school level, before clustering of schools within the intervention group.

C6:How were costs calculated? Line 436 points to the cost of the game, but what are the other costs associated with the intervention (e.g., teacher time)

R6: The intervention game was designed and produced locally. As such it was easy to calculate the amount expended in producing a single game, which was 525 naira only. This cost was then reconverted to USD at the rate of 1 dollar = 350 naira. Other costs such as distribution cost, training cost etc were not computed as it was beyond the objective of the study.

--------------------

**Results**


-Does the analysis presented match the analysis plan?

-Are the results clearly and completely presented?

-Are the figures (Tables, Images) of sufficient quality for clarity?

Reviewer #3:

C7: The analysis approach is not clear, thus the results are not clearly described. I would expect that the main outcome is a risk double difference in the prevalence STH at endline comparing intervention to control, accounting for baseline values. In that way, both time points could be used in the analysis. As it is currently shown, it seems that p values (but no estimates of effect or CIs) are just comparing intervention to control for each round (including baseline). Along those lines, it is not appropriate to only report p values. Risk ratios or odds ratios with 95% CI would be more appropriate. Reliance on p values alone is insufficient for the reader to understand variance.

R7: Thank you very much for the invaluable comments raised here. We have provided the risk ratios and odd ratios (including 95% CI) at the three different time point of evaluation in this study i.e baseline, 3-months and 6-months follow up (Please see table 3, Lines 333-335)

We have also update our text to explain the new tables. Please see lines 317-318, 327-328 and 331-332

C8: The figures (charts) are not particularly useful and would be better represented in tables. Some of the text can be cut in lieu of putting data in the tables.

R9: Yes, figures 3,4,5,6 have been represented as tables as suggested. Please see table 2 (line 319)

--------------------

**Conclusions**


-Are the conclusions supported by the data presented?

-Are the limitations of analysis clearly described?

-Do the authors discuss how these data can be helpful to advance our understanding of the topic under study?

-Is public health relevance addressed?

Reviewer #3:

C10: The conclusions should better address the context as discussed above. More detail on the implications for the use of games and knowledge-based educational campaigns to impact hygiene behavior (and what behaviors were targeted) would be warranted.

R10: We have improved our text to address the concerns raised here. Implications of the use of games to impact hygiene behavior have been added in Line 88-102. More details about cost, delivery and adoption mechanism can also be found between lines 540-552

--------------------

**Editorial and Data Presentation Modifications?**


Use this section for editorial suggestions as well as relatively minor modifications of existing data that would enhance clarity. If the only modifications needed are minor and/or editorial, you may wish to recommend “Minor Revision” or “Accept”.


Reviewer #3:

C11: There is some need for grammar and english language editing to enhance clarity.

R11: We have improved our text to enhance clarity.
--------------------

**Summary and General Comments**


Use this section to provide overall comments, discuss strengths/weaknesses of the study, novelty, significance, general execution and scholarship. You may also include additional comments for the author, including concerns about dual publication, research ethics, or publication ethics. If requesting major revision, please articulate the new experiments that are needed.


Reviewer #3:

C12: With RCTs, it is common to submit pre-analysis plan to a database such as CinicalTrials.org prior to the study. Was this done?

R12: Unfortunately, we didn’t do that. Thank you for this comment. We will take this into consideration henceforth

C13:Is this game really trade marked, as indicated by the name Worms and Ladders"TM"? If so, who owns the trademark and is this something that needs to be disclosed as a conflict of interest?

R13: Yes, the game is trade marked. It’s the intellectual property of the first and last authors. There is no conflict of interest.

C14: How was the game developed? Was it piloted? How were messages decided upon? Did the researchers rely on any behavioral theory in developing the intervention?

R14: We have provided more information to address all the questions raised here in between lines 190-231

**Designing the “Worms and LaddersTM” game**

As an initial step during the development of an intervention tool, a consultation meeting among investigators was held to identify risk factors for STH infections. The risk factors for STH were considered through desk-review of previous epidemiological studies conducted among school-aged children. Since studies have shown that poor sanitary conditions and personal hygiene such as not wearing shoes or slippers, indiscriminate defecation, not washing hands before eating and/or after using the toilet, lack of toilet facilities and dirty environment predisposes children to STH infections (Bethony *et al*., 2006; Ekpo *et al*., 2008; Ekpo *et al*., 2012; Halpenny *et al*., 2013). The health educational messages were then formed in response to each of the identified risk factor. The messages were presented in simple forms to ensure proper understanding. Ten major health educational messages developed were; washing of hands before eating, washing hands with soap after using the toilet, not playing with soil, wearing of slippers or shoes when going outside, avoiding open (indiscriminate) defecation, washing of fruits and vegetables before consumption, covering of food from flies, keeping fingernails clean and cut, not picking food from the ground, and cleaning the environment.

Furthermore, since the target population for the tool is school-aged children, the team adopted the use of visual aids, as it is one of the strongest tool used in communicating messages; particularly when accompanied with interactive methods (Yazachew and Alem, 2004). In addition, the team considered deploying this tool in a way that facilitates group discussion and demonstration. In consideration of all these, a board game was selected as a means to deliver the health education messages to the school children. Therefore, a board game named “Worms and Ladders^TM^” was developed to teach the school-aged children about transmission, symptoms, treatment, prevention and control of STH infections. The game was piloted in a school setting to identify areas that needs to be worked upon to enhance clarity and facilitate understanding. The pilot study was implemented in an area different from where the study schools were selected.

The design of the “Worms and Ladders^TM^” health educational board game follows the popular “Snakes and Ladders” game, based on the concept of reward for good health behavior by moving up a ladder and punishment for risky health behavior by being bitten by the STH worms. Graphic design was done in CorelDraw X13 software. Illustrated pictorials, which explain the characters were used in the design of the game for easy understanding and relativity even to a layman thus improving the all-round development and the intellect of the average child.. The game consists of squares numbered from 1 to 100. Play starts from square number one, and ends at the hundredth square, which is “worm free”. The anterior (head) end of the worms are placed on squares inscribed with statements such as unhygienic behaviors, poor sanitary conditions, which predispose school children to STH infections and transmission. However, the posterior (tail) end of the worm falls on statements that depicts the consequences of such unhygienic behavior or sanitary conditions. On the other hand, the foot of the ladders is placed strategically on the hygienic behaviours (preventive measures) that prevent infection STH. The game can be played by a minimum of 2 and a maximum of 4 persons, to allow proper interaction and assimilation of the health education messages and warnings on the board, using dice and counters. The game starts when a player throws a 1 with a dice. A Player moves up when he/she gets to the foot of a ladder and goes down when he gets to the head of the STH worm. Players are self-guided by the health messages on each square of the board. The players count the number shown on the dice when thrown and the first player to get to the square marked 100 wins the game.

C15:In the introduction, more discussion is needed on the evidence of education on behavior change related to hygiene in general, and STH prevention specifically. Additional literature on the use of games for behavior change would be useful.

R15: We have provided this information between lines 88-102

Health education strategy is economical as it reduces the cost of deworming, increases the level of overall health knowledge and acceptability of deworming interventions (Luong, 2003; Albonico *et al*., 2006) and it also enhances increased understanding of effective health practices (Hotez *et al*., 2006). The inclusion of a health education strategy into school-based deworming programmes has been recommended based on the rationale that exposure to STH infections would be reduced, and re-infection delayed through improved knowledge and behavioural changes (WHO, 2003; Mascarini-Serra, 2011). There is plethora of evidence reinforcing the aforementioned theory on the significant role health educational activities have on positive behavioural change [15-18]. For instance, Bieri and colleagues [13] in China used a video-based health education package to prevent new STH infections after treatment. Similarly, Ejike and colleagues [14] in Nigeria also reported significant improvement in the knowledge, attitudes and practices as regards risk behaviour, prevention and control of schistosomiasis among children who played a health educational board game. Health education thus plays a major role in helminths disease prevention, by decreasing exposure risk through behavioural change, and in increasing health-seeking behaviour.

--------------------

PLOS authors have the option to publish the peer review history of their article ([what does this mean?](https://journals.plos.org/plosntds/s/editorial-and-peer-review-process#loc-peer-review-history)). If published, this will include your full peer review and any attached files.

If you choose “no”, your identity will remain anonymous but your review may still be made public.

**Do you want your identity to be public for this peer review?** For information about this choice, including consent withdrawal, please see our [Privacy Policy](https://www.plos.org/privacy-policy).
Reviewer #3: No

_________________________________________
In compliance with data protection regulations, you may request that we remove your personal registration details at any time. (Use the following URL: https://www.editorialmanager.com/pntd/login.asp?a=r). Please contact the publication office if you have any questions.
