# Supplementary material for: The impact of Worms and Ladders, an innovative health educational board game on Soil-Transmitted Helminthiasis control in Abeokuta, Southwest Nigeria
Source: PLoS Negl Trop Dis. 2020 Sep 25;14(9):e0008486. doi: 10.1371/journal.pntd.0008486 (PMC7549763; doi:10.1371/journal.pntd.0008486)
Supplement: Attachment — Submitted filename: Response to reviewer 2.docx [file pntd.0008486.s003.docx]

Reviewer's Responses to Questions

Reviewer 2:

C1: It is my second revision of this manuscript. My comments on this version methods are:- I suggest to change the term for the game from "treatment" to "intervention".

R1: The main manuscript has been revised. The term "treatment" has been changed to "intervention" accordingly. Thank you

C2: The authors states that schools were "blindly assigned" but not sure if it was systemically or randomly assigned.

R2: Yes one of the researcher (blind) who did not take part in the selection procedures made the randomizing and the selection of schools from the intervention and control clusters respectively. We have restructured the statement to make it clearer to read

Line 121-134: The intervention schools were then selected from Cluster 1 (C1) with a total of 21 schools; and the control schools were selected from cluster 2 with a total of 28 schools. In order to have an ample sampling frame, six schools were randomly selected, at first using balloting from each cluster. Afterwards, three schools were selected randomly out of the six schools in each clusters using the second round of balloting to minimize the cofounding effect that may arise due to cross-communication among schools that shares boundary or are near each other [24]. The final randomization of participating schools to intervention was carried out by one of the researchers (blinded) who did not partake in the schools' selection procedures. Preliminary visits were made to selected schools to document cofounding factors such as the presence of UNICEF-assisted school-based WASH intervention programmes. Schools with UNICEF-assisted programme were replaced purposively with one of the three remaining schools in the sampling frame. The schools in cluster 1 (intervention group) received the newly designed Worms and Ladders^™^ game (Figure 2) while those in cluster 2 (control group) received the common Snakes and Ladders game (Figure 3).

C3: - Lines 180, I am not sure if authors were meaning that an assent form was completed by children older than 16 years old.

R3: Yes. Apart from parental consent, consent forms were completed by children above 16 years, while assent forms were completed by children younger than16 years.

C4: They calculate odds ratio but they do not adjust for confounders that could be interferring the risk.

R4: The confounder we had in our mind was the access to UNICEF-assisted school-based WASH intervention programmes This confounder was removed purposively by replacing such schools in the final selection of the six participating schools. We did not control for other cofounders. All the participating school children are living within similar socioeconomic areas.

Line 129-132: Preliminary visits were made to selected schools to document cofounding factors such as the presence of UNICEF-assisted WASH intervention programmes. Schools with UNICEF-assisted programme were replaced purposively with one of the three remaining schools in the sampling frame.

--------------------
Results

C5: Prevalence is double in the control group, so they are not comparable.

R5: Thank you for this comment. Although the prevalence were incomparable at baseline, it is worthy to note that all participants were dewormed and monitored for egg output. Only those who had no egg output were recruited into the study.

Line 242-248: Following baseline assessments, all the children including the selected participants in the selected schools were dewormed with 400mg Albendazole (single dose). Deworming was carried out by State STH control programmers according to WHO guideline. To verify, that the selected study participants were not infected, egg-output were monitored for six days after deworming. Only non-infected children in intervention and control schools were allowed to participate further in intervention phase of the study.

--------------------

Conclusions

Reviewer #2: -

--------------------
Editorial and Data Presentation Modifications?

Reviewer #2: -

--------------------
Summary and General Comments:

C6: Some of the comments were already done in the last revision but were not changed.

R6: Thank you very much for your comments and inputs. We have looked through the previous revision and have effected the changes. More importantly, the name of the game from treatment to intervention
--------------------
